# Conformation-Dependent High-Affinity Potent Ricin-Neutralizing Monoclonal Antibodies

**DOI:** 10.1155/2013/471346

**Published:** 2012-12-26

**Authors:** Wei-Gang Hu, Junfei Yin, Damon Chau, Charles Chen Hu, Dustin Lillico, Justin Yu, Laurel M. Negrych, John W. Cherwonogrodzky

**Affiliations:** Defence Research and Development Canada-Suffield, P.O. Box 4000, Station Main, Medicine Hat, AB, Canada T1A 8K6

## Abstract

Ricin is a potential biothreat agent with no approved antidote available for ricin poisoning. The aim of this study was to develop potent antibody-based antiricin antidotes. Four strong ricin resistant hybridoma clones secreting antiricin monoclonal antibodies (mAbs) were developed. All four mAbs are bound to conformational epitopes of ricin toxin B (RTB) with high affinity (*K*_*D*_ values from 2.55 to 36.27 nM). RTB not only triggers cellular uptake of ricin, but also facilitates transport of the ricin toxin A (RTA) from the endoplasmic reticulum to the cytosol, where RTA exerts its toxic activity. The four mAbs were found to have potent ricin-neutralizing capacities and synergistic effects among them as determined by an *in vitro* neutralization assay. *In vivo* protection assay demonstrated that all four mAbs had strong efficacy against ricin challenges. D9 was found to be exceptionally effective. Intraperitoneal (i.p.) administration of D9, at a dose of 5 **μ**g, 6 weeks before or 6 hours after an i.p. challenge with 5 × LD50 of ricin was able to protect or rescue 100% of the mice, indicating that mAb D9 is an excellent candidate to be developed as a potent antidote against ricin poisoning for both prophylactic and therapeutic purposes.

## 1. Introduction

Ricin is a 60–65 kDa glycoprotein derived from beans of the castor plant [1]. It consists of a ricin toxin A (RTA) protein and a ricin toxin B (RTB) protein linked by a disulfide bond. RTB binds to galactose residues on the mammalian cell surfaces not only triggering cellular uptake of ricin [2], but also facilitating transport of the RTA from the endoplasmic reticulum (ER) to the cytosol [3, 4], where RTA then enzymatically cleaves ribosomal RNA to stop protein synthesis [5]. Ricin is a highly potent toxin known for humans [6]. Although never laboratory confirmed, ricin was most likely the etiologic agent used in the assassination of Georgi Markov in 1978, demonstrating the extreme lethality of the toxin [7]. Due to its ease of production, worldwide availability, relative stability, and extreme lethality, ricin is listed as a Category B threat agent by Centers for Disease Control and Prevention (Atlanta, USA). The most recent examples of the ricin threat were in August and November 2011, when the New York Times reported that Al-Qaeda was trying to produce ricin bombs for attacks against the United States [8] and the Washington Post reported that four Americans were arrested in connection with an alleged ricin terrorist plot [9]. A ricin terrorist attack is possible if not probable. Unfortunately, there is currently no approved antidote or vaccine available against ricin.

The development of antidotes or vaccines against ricin has proven elusive. Chemical inhibitors targeting ricin have been developed, but these are limited by the high amounts needed for short-term effects and their own toxicity [10–12]. Development of vaccines against ricin has been ongoing, but the efficacy of the most promising candidate, an attenuated derivative of RTA, remains problematic in clinical trials. There was no statistical correlation between serum antiricin antibody titres and *in vitro* ricin-neutralizing activity [13], possibly because the ricin-elicited antibodies were a mixture of neutralizing, nonneutralizing, and even toxin-enhancing antibodies [14, 15] and the antibody composition to the RTA vaccine varies among different individuals. Of the different approaches for medical countermeasures, antiricin antibodies appear the most promising. Much work has been done on developing antibodies, both polyclonal and monoclonal, as antidotes against the toxin. These antibodies were directed against the RTA (blocking its destructive action at the ribosome) [15–22], the RTB (preventing it from binding to and entering the cell) [15, 16, 20, 23, 24], or both [25].

In the present study, to develop potent ricin-neutralizing antibodies, mice were immunized with increasing doses of native ricin, splenocytes were harvested and used to generate hybridoma, and these cells were then cloned, screened, and selected in the medium with ricin [26]. Subsequently, after further characterization by enzyme-linked immunosorbent assay (ELISA) and evaluation by neutralization assays, four ricin-neutralization hybridoma clones were identified. All four monoclonal antibodies (mAbs) were specific to RTB. They were found to have potent ricin-neutralizing capacities and synergistic effects among them as determined by *in vitro* neutralization assay. *In vivo* postexposure protection assay demonstrated that all four mAbs had strong efficacy against ricin challenges *in vivo*. D9 was found to be exceptionally effective. Intraperitoneal (i.p.) administration of D9, at a dose of 5 *μ*g, 6 hours after an i.p. challenge with 5 × LD50 of ricin was found to rescue 100% of the mice. D9 was further evaluated for preexposure prophylaxis against ricin *in vivo*, and 5 *μ*g per mouse delivered by the i.p. route 6 weeks before i.p. challenge of ricin (5 × LD50) protected 100% of the mice. These results indicate that mAb D9 is an excellent candidate to be developed as a potent antidote against ricin poisoning for both prophylactic and therapeutic purposes. 

## 2. Materials and Methods

### 2.1. Animals

Female Balb/c mice (6 week old, 20–25 g) were obtained from the pathogen-free mouse-breeding colony at Defence Research and Development Canada (DRDC)-Suffield, with the original breeding pairs purchased from Charles River Canada (St Constant, QC). 

All mouse experiments were performed in strict accordance with the guidelines set out by the Canadian Council on Animal Care (CCAC). The animal care protocol was reviewed and approved by the Committee on the Ethics of Animal Experiments of DRDC-Suffield (protocol number: J1C-10-1-1-0). All efforts were made to minimize suffering.

### 2.2. Preparation of Ricin Stock

Ricin was prepared from castor beans in DRDC-Suffield. Our caster beans from India weighed 300 mg each and in extraction yields of ricin were 1%. The toxicity of ricin stock was also determined. One LD50 of ricin for mice was determined by the i.p. injection of a series of ricin dilution into mice. The mice were observed for 7 days. The amount of ricin for 1 × LD50 delivered by the i.p. route for one mouse was 0.215 *μ*g; 5 × LD50 was 1.075 *μ*g, which was around 0.03% of a bean. For 5 × LD50 of ricin delivered by the i.p. route, mice died within 2 days. All research with ricin was conducted in a secure biosafety level 2 area and under the preview of the Organisation for the Prohibition of Chemical Weapons (OPCW).

### 2.3. Immunization Scheme

Groups of 5 Balb/c mice were injected by the i.p. route with increasing amounts of ricin (0.2, 1, 5, and 25 × LD50) in 0.1 mL sterile 0.9% saline per mouse. Injections of increasing ricin amounts were 2-3 weeks apart. Two weeks after the final dose, the mice were bled, sera collected, and an ELISA (see below) was used to determine the antiricin IgG antibody titres in sera. 

### 2.4. ELISA

ELISA was performed to evaluate antiricin mAbs. Ninety-six-well Nunc Maxisorp ELISA plates (Canadian Life Technologies, Burlington, ON) were coated with 100 *μ*L per well of 2.5 *μ*g/mL ricin in carbonate bicarbonate buffer, pH 9.6, then incubated overnight at 4°C. After blocking with SuperBlock blocking buffer (Fisher Canada, Nepean, ON), the plates were incubated with 100 *μ*L of serum dilutions or culture supernatants for 2 hours at room temperature. Antiricin mAbs were detected by incubation with 1 : 3,000 diluted horseradish peroxidase- (HRP-) goat anti-mouse IgG (Jackson ImmunoResearch, West Grove, PA, USA) followed by the addition of tetramethylbenzidine (TMB) (Kirkegaard and Perry Laboratories, Gathersburg, MD, USA). Absorbance was measured at 615 nm by a microplate autoreader (Molecular Devices, Sunnyvale, CA, USA).

### 2.5. Generation and Selection of Hybridomas

The two mice with the highest ELISA titres were sacrificed three days after the last booster to collect spleens. The spleens were aseptically dissected, and single splenocyte suspension was prepared [27]. Hybridomas were generated by fusing the splenocytes with Sp 2/0 myeloma cells (ATCC accession number CRL-1581, ATCC, Rockville, MD, USA) using a Clonacell-HY kit (StemCell Technologies, Vancouver, BC), following the manufacturer's instruction and growing these in semisolid medium with 2.5 ng/mL ricin (10 × hybridoma cell culture lethal dose). After 2 weeks of incubation, single-hybridoma clones were picked up from semisolid medium, transferred to 96-well tissue culture plates (Costar, Corning, NY, USA), and then grown for 1 week in Clonacell Medium E with 5 ng/mL ricin (20 × hybridoma cell culture lethal dose) for further selection. The supernatants were removed and assessed by ELISA for antiricin antibodies.

### 2.6. Antibody Purification

The hybridoma clones surviving two rounds of ricin poisoning and secreting antiricin antibodies were expanded. MAbs were purified from the cell culture supernatant by a Melon Gel purification kit (Pierce, Rockford, IL, USA) according to the manufacturer's protocol. The supernatant was dialyzed for two exchanges (1 hour each) in Melon Gel IgG Purification Buffer pH 7.0 and then was added to a column containing the Melon Gel resin. After 5 minute-incubation with end-over-end mixing, the purified IgG was collected in the flowthrough. All IgG purified samples were aliquoted and stored at −20°C. The concentration of antibody was determined by an Easy-Titer Mouse IgG Assay Kit (Pierce) according to the manufacturer's protocol. 

### 2.7. Isotype Determination

All the purified antiricin mAbs were isotyped using a mouse IsoStrip Kit (Roche Diagnostics, Laval, QC) following the manufacturer's instruction.

### 2.8. Western Blot Analysis

Ricin, RTA, or RTB (Sigma-Aldrich, Oakville, ON) was boiled for 10 min in Laemmli sample buffer (with or without 5%  *β*-mercaptoethanol), then separated by 10% NuPAGE Bis-Tris gels (Invitrogen, Burlington, ON), and transferred onto nitrocellulose membranes using an XCell SureLock Novex Mini-Cell System (Invitrogen). The membranes were then blocked with 3% (w/v) bovine serum albumin in Tris buffer saline containing 0.05% tween-20 (TBST) for 1 hour and probed with antiricin mAbs, 1 : 1,000 in TBST overnight at 4°C. After washing three times with TBST at room temperature, the membranes were incubated for 1 hour with 1 : 3,000 diluted HRP-goat anti-mouse IgG (Caltag Laboratories). Membranes were washed three times in TBST and developed with enhanced chemiluminescent (ECL) reagent (Millipore, Billerica, MA, USA). The image was recorded using a VersaDoc 5000 MP imagining system (Bio-Rad). SDS-PAGE gels were visualized by SimplyBlue Safestain staining (Invitrogen) and the molecular weights of samples were estimated by comparison to the relative mobility values of standards of known molecular weight. The image of the stained SDS-PAGE gel was recorded using the VersaDoc 5000 MP imagining system.

### 2.9. Affinity Analysis

The affinities for mAbs binding to ricin were determined using a Surface Plasmon Resonance (SPR) biosensor, SensiQ Pioneer (ICx Technologies, Oklahoma, OK, USA). Briefly, ricin (10 *μ*g/mL) diluted in 10 mM acetate buffer pH 4.5 was first immobilized onto the COOH1 chip following the standard 1-ethyl-3-(3-dimethylpropyl)-carbodiimide (EDC) plus N-hydroxysuccinimide (NHS) (Sigma-Aldrich) coupling chemistry and 250 response units (RU) of ricin were immobilized. The system was operated at 25°C. Kinetic measurements were carried out by 2 min injection at a flow rate of 25 *μ*L/min of serial dilutions of each mAb from 2 to 250 nM in 4-(2-hydroxyethyl)-1-piperazineethanesulfonic acid (HEPES-) buffered saline containing 3 mM ethylenediaminetetraacetic acid (EDTA), 150 mM NaCl and 0.005% tween-20, and dissociation for 6 min. The ricin immobilized chip surface was regenerated by injection of 10 mM phosphoric acid for 120 sec after each cycle. The data of dissociation (*k*_*off*⁡_) and association (*k*_*on*⁡_) rate constants were obtained with the SensiQ Qdat software, corrected by subtraction of the zero antibody concentration flow cell as well as zero ricin flow cell; values for the apparent equilibrium dissociation constant (*K*_*D*_) were calculated from the ratio of *k*_*off*⁡_ and *k*_*on*⁡_. 

### 2.10. *In Vitro* Neutralization Assay

A Vero cell (ATCC, Burlington, ON) toxicity neutralization assay with Alamar blue as an indicator was performed. Ricin was incubated with a serial dilution of each mAb for 2 hours at 37°C in 96-well plates. Ten thousand Vero cells cultured in 50 *μ*L of DMEM with 10% fetal bovine serum (Hyclone, Fisher Canada) were added into the mixture. The final volume of the cell mixture was 200 *μ*L with ricin concentration of 7.5 ng/mL, mAb concentrations from 5 *μ*g/mL to 2 ng/mL. After incubation at 37°C, 5% CO_2_ for 2 days, 20 *μ*L of Alamar blue (TREK Diagnostic System, Cleveland, OH, USA) was added to each well and the plate was incubated for 6-7 hours. On a plate reader (Molecular Devices), the plate was read at an absorbance of 570 nm with 600 nm as a reference wavelength. Readings were normalized by subtracting the absorbance reading of wells without cells. The cell viability was expressed as cell survival ratio relative to the control without ricin (Vero cells plus mAbs).

### 2.11. *In Vivo* Protection Assay

For postexposure therapeutic efficacy study, groups of 4–8 mice were given 5 × LD50 of ricin per mouse by the i.p. route and then 5 *μ*g of mAb per mouse was administered by the i.p. route to mice at 1, 2, 4, 6, or 8 hours after ricin poisoning. For preexposure prophylactic efficacy study, 5 *μ*g of mAb was administered by the i.p. route to each mouse (group of 4) and the mice were poisoned by the i.p. route with 5 × LD50 of ricin per mouse at 1, 2, 4, 6, or 8 weeks after administration of mAb. The mice were observed for morbidity and mortality over one week.

### 2.12. Determination of D9 Half-Life in Serum

To evaluate the half-life of D9 in serum, groups of 4 mice were injected by the i.p. route with 5 *μ*g/mouse of D9 in 100 *μ*L PBS and were bled from a superficial tail vein at 1, 7, 14, and 23 days after injection. D9 concentrations in sera were then measured by the ELISA over time, expressed as percentages of the D9 concentration in sera on day 1, and then plotted against time in days after treatment.

### 2.13. Statistical Analysis

For* in vitro* neutralization assays, normalized absorbance readings were analysed for statistical significance using the Student's *t*-test.

## 3. Results

### 3.1. Generation and Selection of Ricin Resistant Hybridoma Clones

Mice were immunized with a stepwise increase of the ricin amount (0.2, 1, 5, and 25 × LD50 per mouse). Following the last booster, the two mice with the highest ELISA antiricin titers were sacrificed and splenocytes were prepared and fused with myeloma cells in a standard hybridoma fusion protocol. After growth in semisolid medium plus 2.5 ng/mL ricin, and subsequent culture in liquid medium with 5 ng/mL ricin, 25 hybridoma clones survived this high concentration of ricin poisoning culture. 

### 3.2. Screening of Antiricin Neutralizing mAbs *In Vitro*

**Figure 1:**
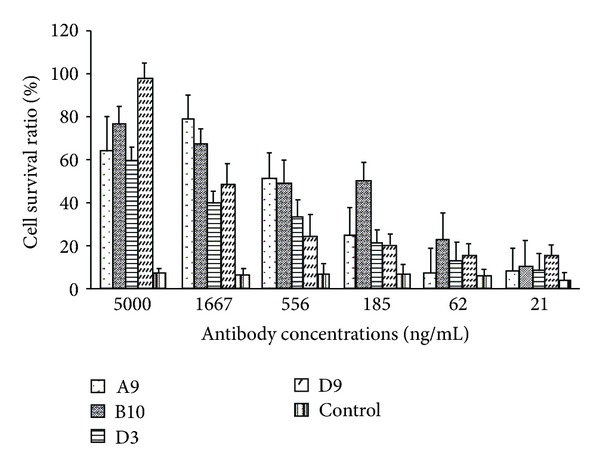
*In vitro* neutralization assay. Thirty-five ng/mL of ricin were preincubated with a serial dilution of each mAb for 2 hours and then exposed to 10^4^ Vero cells/well for 2 days before evaluation of cell viability using Alamar blue staining. Data are means of triplets.

Twenty out 25 ricin resistant hybridoma clones were found to secret mAbs reactive to ricin in ELISA. A Vero cell toxicity neutralization assay with Alamar blue as an indicator was performed to screen for antiricin neutralizing mAbs from the 20 clones secreting antiricin antibodies. Thirty-five ng/mL of ricin were incubated with a serial dilution of each mAb (from 5 *μ*g/mL to 2 ng/mL at threefold dilution) for 2 hours and then 10^4^ Vero cells/well were added into the mixture. After culture for 2 days, Alamar blue was added to evaluate viability of the Vero cells. Twelve clones showed neutralizing titer against ricin. The four hybridoma clones (A9, B10, D3, and D9) were chosen out of 12 clones, having the highest neutralizing titers (*P* < 0.01), as shown in Figure 1.

### 3.3. Characterization of the Four mAbs

**Figure 2:**
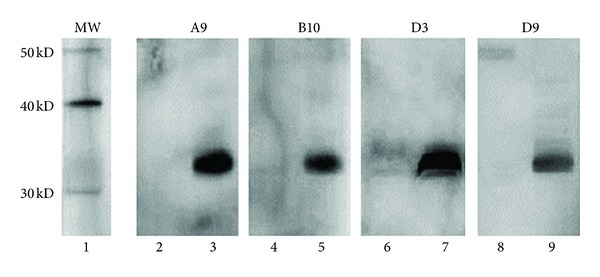
Western blot analysis of mAb ricin binding activity. RTB (0.4 *μ*g, each lane) was migrated by 10% SDS-PAGE in reducing (lanes 2, 4, 6, and 8) or nonreducing (lanes 3, 5, 7, and 9) condition and transferred to nitrocellulose membrane. Membranes were probed with each mAb, respectively, and followed by antimouse antibody-HRP before finally being developed with ECL kit. The image was recorded using the VersaDoc 5000 MP imagining system. Lane 1 is molecular weight marker.

In order to determine which subunit of the ricin the four mAbs bound to, Western blot analysis with RTA or RTB was performed. All four mAbs only bound to RTB, not RTA. Interestingly, when RTB was reduced, no mAb bounds to this subunit as shown in Figure 2. In the RTB molecule, there are four intrachain disulfide bridges, which hold RTB into two globular domains [28]. Each domain is stabilized by two disulfide bridges. When the four intrachain disulfide bridges in RTB were broken by 2-mercaptoethanol, no mAb could bind to it, indicating that the epitopes on RTB recognized by all four mAbs are conformational. 

All four antiricin neutralizing mAbs were isotyped using a mouse IsoStrip kit and all the mAbs showed the same subtype of heavy chain, gamma 1, and the same type of light chain, kappa.

To characterize the binding affinity of the four antiricin neutralizing mAbs, a SPR biosensor was used. Ricin was captured onto a biosensor chip, various concentrations of mAbs were passed through the flow cell, and the binding kinetics were recorded. The kinetic rate constants *k*_*on*⁡_ and *k*_*off*⁡_ were calculated from the ascending rate of resonance units during association and the descending rate during dissociation. The *K*_*D*_ of each mAb for ricin was determined from the ratio of *k*_*off*⁡_/*k*_*on*⁡_. As shown in Table 1, mAbs D9 and D3 had relatively high affinity to ricin with *K*_*D*_s of 2.55 and 2.88 nM, while B10 and A9 had relatively low affinity to ricin with *K*_*D*_s of 21.37 and 36.27 nM.

### 3.4. Synergistic Effect among the Four mAbs

**Figure 3:**
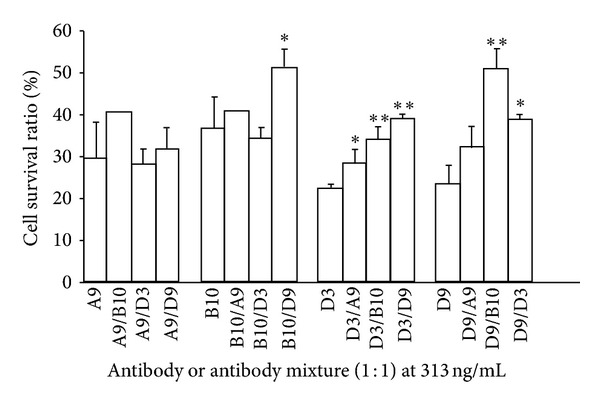
Synergistic effect of mAbs to neutralize ricin *in vitro*. mAbs or mAb mixtures (1 : 1 ratio) at concentration of 313 ng/mL were premixed with ricin 35 ng/mL for 2 hours before exposure to Vero cells. Cell viability was assessed by Alamar blue staining. Data are means of triplets. ∗ or ∗∗ indicate *P* < 0.05 or 0.01, as compared with the single antibody control group.

Combinations of the different mAbs were assessed by an *in vitro* neutralization assay to evaluate the synergism of the mAbs in neutralization of ricin. Pairs of mAbs (1 : 1 ratio) at a final concentration of 313 ng/mL were evaluated. As shown in Figure 3, synergistic effects were observed, especially for D9 and B10. It should be noted that the synergistic effects were identified by using half the amount of each antibody (e.g., 156 ng/mL) as for the single mAb by itself (e.g., 313 ng/mL). For example, the value for the pair of B10 and D9 was higher than B10 or D9 itself (*P* < 0.05 or *P* < 0.01).

### 3.5. Protective Efficacies of the Four mAbs against Ricin in Mice

**Figure 4:**
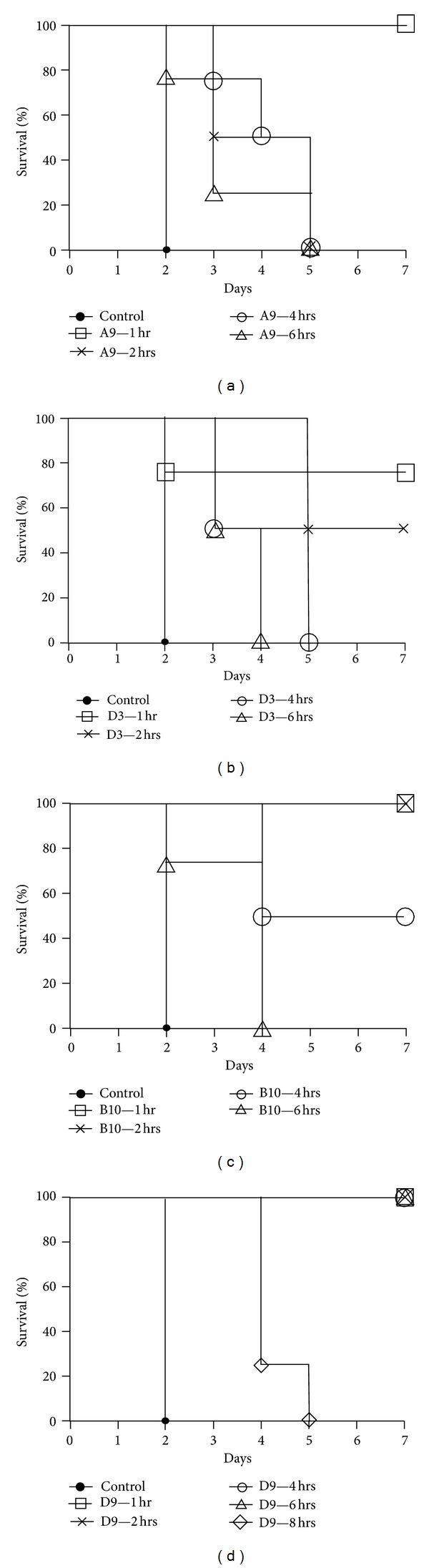
*In vivo* postexposure therapy assay. Ricin was given at the dose of 5 × LD50 to mice by i.p. route. Each mAb at the dose of 5 *μ*g was administered i.p. at 1, 2, 4, 6, or 8 hours after ricin challenge and then mouse survival rate was monitored for 7 days.

A mouse model was applied in order to evaluate the protective efficacies of all four antiricin neutralizing mAbs in a postexposure setting. Ricin was given at the dose of 5 × LD50 to mice by i.p route. Each mAb at the dose of 5 *μ*g was administered by the i.p. route at 1, 2, 4, 6, or 8 hours after ricin challenge. MAbs showed different therapeutic efficacy shown in Figure 4. D9 could rescue mice up to 6 hours after challenge, allowing 100% mice survival, while D3 showed only 75% protection at 1 hour after challenge. When given to mice at 8 hours after challenge, D9 could extend mouse lives to 4-5 days as compared to survival time of 2 days in untreated controls. The therapeutic efficacies for A9 and B10 were in the middle between D9 and D3. A9 could rescue 100% mice at 1 hour after challenge, while B10 up to 2 hours after challenge.

**Figure 5:**
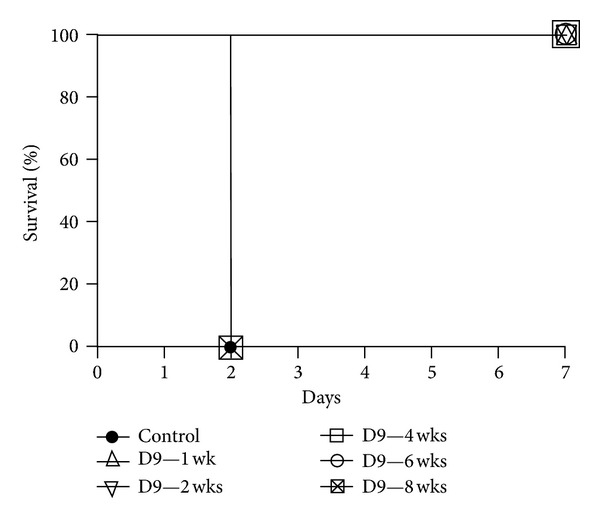
*In vivo* preexposure prophylaxis assay. D9 mAb at the dose of 5 *μ*g was administered by the i.p. route into mice at 1, 2, 4, 6, or 8 weeks before ricin challenge (5 × LD50) by the i.p. route and then mouse survival rate was monitored for 7 days.

Study of the preexposure prophylaxis by antiricin neutralizing mAbs is relevant for individuals in high-risk groups assigned a mission in the ricin-contaminated zone. In the literature, antiricin neutralizing antibodies can be given to 24 hours before ricin poisoning to protect mice [17, 18]. From our work, no death was observed when 5 *μ*g of D9 was given at 1, 2, 4, and 6 weeks before mice were challenged with 5 × LD50 ricin (Figure 5).

**Figure 6:**
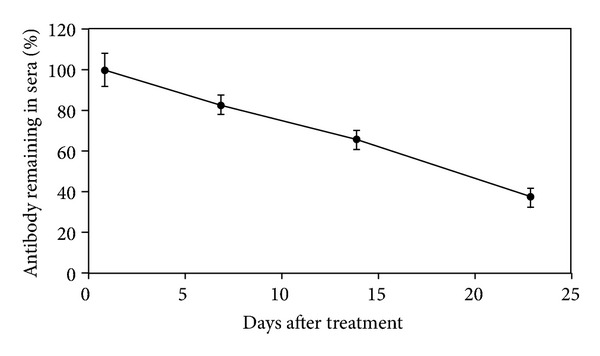
Half-life of D9 in mouse serum. D9 mAb at the dose of 5 *μ*g was administered by the i.p. route into mice. Sera were collected at different time points to calculate plasma concentrations of D9 using an immunoassay. The D9 remaining in sera is expressed as percentages plotted against time in days on the figure.

In order to evaluate the half life of D9 in mice, pharmacokinetic study was performed. After i.p. injection of 5 *μ*g D9 in mice, serum concentrations of D9 were determined by antiricin ELISA. As shown in Figure 6, a gradual decrease of D9 in plasma was observed over time. The half-life of D9 in mice was estimated at 18.5 days. 

## 4. Discussion

There are two major groups of antidotes, antibodies and chemical compounds. The history of using antibodies as effective antidotes against toxins can be traced back to 1890 [29], when antiserum from a tetanus-immune animal protected tetanus toxin-mediated mortality of naïve animals. Since then, antibodies have played a pivotal role in neutralizing toxins [30, 31]. There are several advantages for antibodies as antidotes as compared to the chemical antidotes [32–34]. Firstly, antibodies have a long half-life in the body. Secondly, antibodies are natural and nontoxic products. Lastly, current biotechnology allows the development of antibodies possessing a defined specificity against most toxins.

In order to develop highly potent antiricin neutralizing mAbs, mice have been needed to be immunized by an immunogenic dose of ricin, typically 5 *μ*g per mouse. However, 5 *μ*g ricin is equivalent to 25 × LD50 if administered as a primary immunization dose to the mouse. We have observed that mice could survive a large dose of ricin poisoning if the mice were poisoned by a stepwise increase of the ricin dose. In this way, mice were immunized by the i.p. route with ricin from 0.2 × LD50 to 25 × LD50 and a high antiricin antibody titer was obtained (data not shown). 

The production of mAbs using hybridoma technology was invented by George Köhler and César Milstein in 1975 [35]. Since then, this hybridoma technology has been improved greatly by different novel methods for fusing, growing, selecting, cloning, and screening hybridoma clones. Even so, the selection, cloning, and screening are often regarded as the main bottleneck in the development of effective hybridoma clones for antibodies. In our study, we combined a methylcellulose-based semisolid hybridoma selective medium with ricin (2.5 ng/mL) for hybridoma selection, cloning, and screening in one single step to arrive at the desired ricin resistant hybridoma clones. In the unique step, single-hybridoma cells were distributed evenly in the semisolid medium and only ricin resistant single-hybridoma cells could grow to form monoclonal colonies. The ricin resistant hybridoma clones were then transferred into 96-well plates for further selection in liquid medium with a higher concentration of ricin (5 ng/mL). As a result, 25 hybridoma clones were resistant to two cycles of ricin poisoning (semisolid and liquid medium). Twenty clones secreted antiricin antibodies and among these, 12 clones secreted antiricin neutralization mAbs. The result is an improvement over traditional approach to develop antiricin neutralizing antibody hybridomas [24, 36]. The best 4 antiricin neutralizing hybridoma clones were selected, all of which were found to be RTB specific. Although RTB itself is not toxic, it plays a pivotal role in ricin toxicosis. RTB binding to galactose residues on the cell surface is involved in not only triggering cellular uptake of ricin [2], but also facilitating transport of RTA from the ER to the cytosol [3, 4], where RTA exerts enzymatic toxicity. Theoretically, RTB is the logical target for neutralizing antibodies, as these would block the entry of ricin into cells and the transportation of RTA to the cytosol. However, it seems to be more difficult to develop anti-RTB neutralizing mAbs than anti-RTA neutralizing mAbs. One of the reasons is that to date the immunodominant epitopes on RTB have been found not to provide neutralizing protection. In other words, RTB is poor in elicit antiricin neutralizing antibodies although it is highly immunogenic in eliciting nonneutralizing antiricin antibodies [24]. To date, only a few anti-RTB neutralizing antibodies have been reported [20, 23, 24, 37, 38]. 

RTB is a galactose-specific lectin with 262 residues folded into two globular domains. Each domain is formed by similar folding topologies via two intrachain disulfide bridges and responsible for binding to one terminal galactose residue on the cell surface [28]. The two galactoside binding pockets are structurally similar and formed by a sharp bend in Asp-Val-Arg tripeptide [26]. The antigen-binding sites of antibodies are much bigger than RTB galactose binding pockets. Interestedly, all our anti-RTB mAbs did not bind to RTB when the four intrachain disulfide bridges in RTB were broken and the domain structures were disturbed, indicating that all four mAbs most likely bind to conformational epitopes on RTB. It is somewhat perplexing how a RTB-specific neutralizing antibody achieves ricin neutralizing function, given that RTB has two galactose binding sites that work independently and are separated by distance [39]. Since the two RTB domains are homologous and structurally similar, it is possible that our RTB-specific mAbs bind to conformational epitopes sharing the resemblance between two domains and covering the galactoside binding pockets so as to block both two galactose binding pockets and then interrupt ricin binding to cells. In addition, another possibility also exists that our mAbs bind to somewhere else but not galactose binding pockets of RTB and then interrupt the transport of RTA from the ER to the cytosol. Nevertheless, these hypotheses need further experiments to confirm.

The *in vivo* protective efficacy for the four mAbs was first evaluated in a coincubation assay. All of them showed the protection of mice against ricin poisoning. The therapeutic efficacy of the four mAbs for postexposure therapy was then examined *in vivo*. The therapeutic efficacy of antiricin antibody-based treatment is largely dependent on timing of administration of rescuing antibody relative to exposure. A relatively wide therapeutic window will provide necessary time for exposed individual to obtain antiricin antibody treatment in the event of a ricin attack. Therefore, the therapeutic window was determined for each antiricin neutralizing mAb. Although all four mAbs showed postexposure therapeutic functions against ricin poisoning, their therapeutic windows were different. When antibody dose was 5 *μ*g, the best was D9, then B10, followed by A9 and D3 in order. The four mAbs were further characterized. They were isotyped using a mouse IsoStrip kit and all the mAbs showed the same subtype of heavy chain, gamma 1, and the same type of light chain, kappa. Their ricin binding affinities were measured by SPR and they had different *K*_*D*_s ranging from 2.55 to 36.27 nM. The highest was D9, then D3, followed by B10 and A9 in order. In addition, synergistic ricin neutralization effects among different mAbs were evaluated by pairs in an *in vitro* cell-based assay. All of these showed synergistic effect when paired with others. Taken together, the four antiricin RTB neutralizing mAbs appeared different. Their different ricin neutralization activities were more related with their epitope specificities than ricin-binding affinities and not related with their antibody isotype. 

Ricin acts very fast and leaves a very short therapeutic window (effective timing of administration of therapeutic antibodies) for postexposure medical countermeasures. The only way to improve the chance of success to rescue subjects from ricin-intoxication is to develop highly potent neutralizing antibody. To date, dozens of antiricin neutralizing mAbs evaluated *in vivo* have been reported and it is hard to compare their therapeutic efficacies [20, 21, 25, 40–42]. There are many factors, which are attributed to the efficacy outcome, such as mouse strains (inbreed, outbreed) [20, 25], sex (male, female) [21, 41] and age (6–8 weeks, 8–12 weeks) [41, 42], rich challenge doses (5 × LD50, 10 × LD50) [25, 41] and routes (i.p., intranasal) [20, 40], mAb compositions (single, cocktail) [20, 25], and routes (i.p., intravenous) [21, 41]. These factors should be taken into account when the therapeutic efficacy among different mAbs from different laboratories is compared. There are several publications regarding antiricin neutralizing mAbs evaluation *in vivo* in a similar setting to ours, i.p. route used for both ricin challenge and antibody administration [17, 25, 40, 41]. The best reported result has been that the administration of 10 *μ*g of antiricin mAb GD12 per mouse as much as 6 hours after ricin challenge (5 × LD50) rescued 100% mice from toxin-induced death over a 3-day period of observation [41] and 5 *μ*g GD12 protected mice 24 hours before the ricin challenge [17]. D9 is twice as potent as GD12 in a postexposure therapeutic setting and much more potent in a preexposure prophylactic setting. 

It is necessary to humanize murine mAbs for clinical applications since these antibodies have a serious problem in humans, which is serum sickness due to foreignness to humans [43]. Currently, therapeutic settings, using antibody-based drugs, require a large dosage (hundreds mg) and multiple doses. As a result, animal antibody's immunogenicity in humans is a critical concern. Repeating administration of these mAbs may result in rapid clearance of the animal antibodies in humans and anaphylaxis, which can sometimes be fatal [44, 45]. Our data demonstrated that only a very little dose of our antibody D9, such as 5 *μ*g per mouse (equivalent to 1.4 mg per person), could rescue 100% mice 6 hours after ricin challenge (5 × LD50) and protected mice 6 weeks before the ricin challenge. Although one administration of mAb D9 is impractical to prophylactically protect the public against ricin for a long period of time, it is practical to prophylactically protect first responders and military personnel to entering ricin-contaminated zones to perform their duties within six weeks. Therefore, D9 is an excellent candidate to be humanized as a potent antidote against ricin poisoning for both prophylactic and therapeutic purposes.

## 5. Conclusions

In the current study, four antiricin mAbs were developed by a unique cloning and selection approach with ricin poisoning. These mAbs bound to conformational epitopes of RTB with high affinity and showed potent ricin-neutralizing and synergistic effects. The best mAb, D9, at a dose of 5 *μ*g, 6 hours after or 6 weeks before 5 × LD50 ricin challenge could rescue or protect 100% of the mice. These results indicate that D9 is an excellent candidate to be humanized as a potent antidote against ricin poisoning for both prophylactic and therapeutic purposes.

## Figures and Tables

**Table 1:** Kinetic constants of antiricin neutralizing mAbs binding to ricin.

mAb	*k*_*on*⁡_ (M^−1^s^−1^) × 10^5^	*k*_*off*⁡_ (s^−1^) × 10^−3^	*K*_*D*_ (nM)
A9	0.68 ± 0.13	2.48 ± 0.78	36.27 ± 3.62
B10	7.02 ± 0.18	14.89 ± 2.83	21.37 ± 3.21
D3	4.83 ± 0.26	1.39 ± 0.09	2.88 ± 0.33
D9	1.84 ± 0.26	0.47 ± 0.08	2.55 ± 0.12
